# 4,4′-[4,4′-Sulfonyl­bis­(*p*-phenyl­ene­oxy)]dibutanoic acid

**DOI:** 10.1107/S1600536811011871

**Published:** 2011-04-07

**Authors:** Chun-Yan Fu, Yong-Hui Liu, Zhong-Liu Zhou, Hong Tang

**Affiliations:** a Shaoyang Medical College Level Specialty School, Department of Pharmacy, Shaoyang, Hunan 422000, People’s Republic of China; b Red Cross Hospital at Xinshao County of Hunan Province, Shaoyang, Hunan 422000, People’s Republic of China; c Chemistry Science and Technology School, Zhanjiang Normal University, Zhanjiang, Guangdong 524048, People’s Republic of China

## Abstract

In the title compound, C_20_H_22_O_8_S, the dihedral angle between the two benzene rings is 81.6 (3)°. The benzene-connected portions of the alk­oxy substituents are almost coplanar with their respective rings [C—C—O—C torsion angles of 174.77 (17) and −178.5 (4)°]. One of the butanoic acid groups is disordered over two conformations with a site-occupancy ratio 0.719 (6):0.281 (6). In the crystal, pairs of O—H⋯O hydrogen bonds link the mol­ecules into infinite zigzag chains along [130].

## Related literature

For bis­phenol S (systematic name 4,4′-sulfonyl­diphenol) as a reactant in ep­oxy reactions and its use in fast-curing ep­oxy resin glues, see: Askarinejad & Morsali (2006 ▶); Danzl *et al.* (2009 ▶); Bashiri *et al.* (2009 ▶). For its use in the manufacture of pharmaceuticals, adhesives, biocides and agricultural products, see: Howard & David (2002 ▶); Howard *et al.* (2005 ▶); Yasue *et al.* (2009 ▶). For synthesis details and a related structure, see: Zheng *et al.* (2007 ▶).
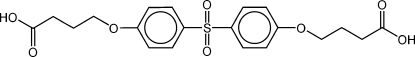


## Experimental

### 

#### Crystal data

C_20_H_22_O_8_S
*M* *_r_* = 422.45
Monoclinic, 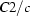

*a* = 30.945 (7) Å
*b* = 8.0964 (18) Å
*c* = 16.032 (3) Å
β = 94.711 (5)°
*V* = 4003.1 (15) Å^3^
*Z* = 8
Mo *K*α radiation
μ = 0.21 mm^−1^
*T* = 296 K
0.20 × 0.18 × 0.12 mm

#### Data collection

Bruker SMART CCD area-detector diffractometer
Absorption correction: multi-scan (*SADABS*; Sheldrick, 1996 ▶) *T*_min_ = 0.959, *T*_max_ = 0.975
12299 measured reflections
4362 independent reflections
2927 reflections with *I* > 2σ(*I*)
*R*_int_ = 0.041

#### Refinement

*R*[*F* ^2^ > 2σ(*F* ^2^)] = 0.045
*wR*(*F* ^2^) = 0.139
*S* = 1.00
4362 reflections
327 parameters
19 restraints
H-atom parameters constrained
Δρ_max_ = 0.30 e Å^−3^
Δρ_min_ = −0.21 e Å^−3^

### 

Data collection: *SMART* (Bruker, 2007 ▶); cell refinement: *SAINT* (Bruker, 2007 ▶); data reduction: *SAINT*; program(s) used to solve structure: *SHELXS97* (Sheldrick, 2008 ▶); program(s) used to refine structure: *SHELXL97* (Sheldrick, 2008 ▶); molecular graphics: *SHELXTL* (Sheldrick, 2008 ▶); software used to prepare material for publication: *SHELXTL*.

## Supplementary Material

Crystal structure: contains datablocks global, I. DOI: 10.1107/S1600536811011871/pk2315sup1.cif

Structure factors: contains datablocks I. DOI: 10.1107/S1600536811011871/pk2315Isup2.hkl

Additional supplementary materials:  crystallographic information; 3D view; checkCIF report

## Figures and Tables

**Table 1:** Hydrogen-bond geometry (Å, °)

*D*—H⋯*A*	*D*—H	H⋯*A*	*D*⋯*A*	*D*—H⋯*A*
O8*B*—H8*B*⋯O2^i^	0.82	2.07	2.890 (11)	180
O8—H8*A*⋯O2^i^	0.82	1.77	2.587 (4)	178
O3—H3⋯O7*B*^ii^	0.82	1.84	2.623 (13)	159
O3—H3⋯O7^ii^	0.82	1.85	2.668 (5)	177

Symmetry codes: (i) 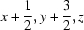; (ii) 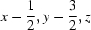.

**Fig. 1.**
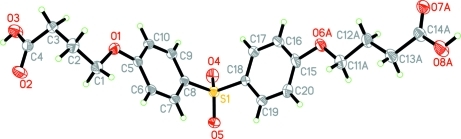
The molecular structure of the title compound, with displacement ellipsoids at the 30% probability level. The disorder in the minor component has been omitted to enhance clarity.

## supplementary crystallographic information

### Comment

Bisphenol S (BPS) is an organic compound with the formula (C_6_H_4_OH)_2_SO_2_.  It has two phenol functional groups on either side of a sulfonyl group. It is commonly used as a reactant in epoxy reactions, and is used in fast-curing epoxy resin glues (Askarinejad & Morsali, 2006; Bashiri *et al.*, 2009; Danzl *et al.*, 2009; Yasue *et al.*, 2009). Bisphenol S is also used in organic synthesis as an organosulfur source in the manufacture of pharmaceuticals, adhesives, biocides and agricultural products (Howard & David, 2002; Howard *et al.*, 2005).  In this article, we present the synthesis and crystal structure of a new potential ligand derived from bisphenol S, which contains multiple oxygen donors and flexible aliphatic spacers.

As shown in Figure 1, the benzene-connected portions of the alkoxy substituents lie almost coplanar with the ring [C–C–O–C torsion angle = 174.77 (17) and -178.5 (4)°, respectively]. The two benzene rings make a dihedral angle of 81.6 (3)°. It is noteworthy that one of the butanoic acid groups is disordered over two components with site occupancy ratio 0.719 (6):0.281 (6).  In the crystal, O—H···O hydrogen bonds link the molecules into a zigzag 1-D infinite chain that propagates along the [1 3 0] direction.  These chains are further interwoven by C—H···O and C—H···π contacts that stabilize the packing.

### Experimental

Reagents and solvents were of commercially available quality. The title complex was synthesized according to the method of Zheng *et al.*, 2007. To a solution of bisphenol S (0.01 mol) in acetonitrile (50 ml), anhydrous potassium carbonate (0.02 mol) and ethyl 4-bromobutanoate (0.01 mol) were mixed. The mixture solution was refluxed for 6 h and filtered. The filtrate was evaporated under reduced pressure and the solid product was dissolved in water/ethanol (1:2 *v*/*v*), then sodium hydroxide (0.02 mol) was added. The solution was refluxed for another 24 h, then acidified with dilute HCl. The crude product was separated by filtration and crystals of the title compound were prepared by recrystallization from a mixture of water and ethanol (1:1).

### Refinement

All H atoms were placed in idealized positions (C—H = 0.93–0.97 Å, O—H = 0.82 Å and refined as riding atoms with *U*_iso_(H) = 1.2*U*_eq_(C) and with *U*_iso_(H) = 1.5*U*_eq_(O). One of the butanoic acid groups is disordered over two conformations with site occupancy ratio 0.719 (6):0.281 (6). All distances in the minor component were restrained to within 0.01 Å of their equivalents in the major component.

### Crystal data

**Table:**

C_20_H_22_O_8_S	*F*(000) = 1776
*M**_r_* = 422.45	*D*_x_ = 1.402 Mg m^−^^3^
Monoclinic, *C*2/*c*	Mo *K*α radiation, λ = 0.71073 Å
Hall symbol: -C 2yc	Cell parameters from 2472 reflections
*a* = 30.945 (7) Å	θ = 2.6–23.6°
*b* = 8.0964 (18) Å	µ = 0.21 mm^−^^1^
*c* = 16.032 (3) Å	*T* = 296 K
β = 94.711 (5)°	Block, colorless
*V* = 4003.1 (15) Å^3^	0.20 × 0.18 × 0.12 mm
*Z* = 8	

### Data collection

**Table:**

Bruker SMART CCD area-detector diffractometer	4362 independent reflections
Radiation source: fine-focus sealed tube	2927 reflections with *I* > 2σ(*I*)
graphite	*R*_int_ = 0.041
φ and ω scans	θ_max_ = 27.1°, θ_min_ = 2.6°
Absorption correction: multi-scan (*SADABS*; Sheldrick, 1996)	*h* = −39→35
*T*_min_ = 0.959, *T*_max_ = 0.975	*k* = −10→8
12299 measured reflections	*l* = −20→20

### Refinement

**Table:**

Refinement on *F*^2^	Primary atom site location: structure-invariant direct methods
Least-squares matrix: full	Secondary atom site location: difference Fourier map
*R*[*F*^2^ > 2σ(*F*^2^)] = 0.045	Hydrogen site location: inferred from neighbouring sites
*wR*(*F*^2^) = 0.139	H-atom parameters constrained
*S* = 1.00	*w* = 1/[σ^2^(*F*_o_^2^) + (0.076*P*)^2^] where *P* = (*F*_o_^2^ + 2*F*_c_^2^)/3
4362 reflections	(Δ/σ)_max_ < 0.001
327 parameters	Δρ_max_ = 0.30 e Å^−^^3^
19 restraints	Δρ_min_ = −0.21 e Å^−^^3^

### Special details

**Table:**

Geometry. All esds (except the esd in the dihedral angle between two l.s. planes) are estimated using the full covariance matrix. The cell esds are taken into account individually in the estimation of esds in distances, angles and torsion angles; correlations between esds in cell parameters are only used when they are defined by crystal symmetry. An approximate (isotropic) treatment of cell esds is used for estimating esds involving l.s. planes.
Refinement. Refinement of F^2^ against ALL reflections. The weighted R-factor wR and goodness of fit S are based on F^2^, conventional R-factors R are based on F, with F set to zero for negative F^2^. The threshold expression of F^2^ > 2σ(F^2^) is used only for calculating R-factors(gt) etc. and is not relevant to the choice of reflections for refinement. R-factors based on F^2^ are statistically about twice as large as those based on F, and R- factors based on ALL data will be even larger.

### Fractional atomic coordinates and isotropic or equivalent isotropic displacement parameters (Å^2^)

**Table:**

	*x*	*y*	*z*	*U*_iso_*/*U*_eq_	Occ. (<1)
S1	0.340925 (17)	0.09320 (6)	0.19395 (3)	0.04130 (18)	
O1	0.21477 (5)	0.09741 (18)	0.44895 (9)	0.0480 (4)	
O2	0.13002 (5)	−0.23795 (19)	0.54170 (11)	0.0623 (5)	
O3	0.08084 (6)	−0.0961 (2)	0.46295 (12)	0.0643 (5)	
H3	0.0708	−0.1887	0.4537	0.096*	
O4	0.31868 (5)	0.1453 (2)	0.11609 (9)	0.0593 (5)	
O5	0.36503 (5)	−0.05895 (19)	0.19703 (11)	0.0557 (4)	
C1	0.21744 (7)	−0.0104 (3)	0.52061 (13)	0.0467 (5)	
H1A	0.2459	−0.0025	0.5507	0.056*	
H1B	0.2126	−0.1240	0.5031	0.056*	
C2	0.18298 (7)	0.0436 (3)	0.57577 (14)	0.0512 (6)	
H2A	0.1909	0.1511	0.5990	0.061*	
H2B	0.1826	−0.0333	0.6221	0.061*	
C3	0.13776 (7)	0.0545 (3)	0.53276 (15)	0.0462 (5)	
H3A	0.1198	0.1173	0.5682	0.055*	
H3B	0.1391	0.1158	0.4811	0.055*	
C4	0.11608 (7)	−0.1071 (3)	0.51281 (13)	0.0425 (5)	
C5	0.24467 (6)	0.0837 (2)	0.39240 (13)	0.0374 (5)	
C6	0.28269 (6)	−0.0081 (2)	0.40364 (13)	0.0404 (5)	
H6	0.2888	−0.0694	0.4522	0.049*	
C7	0.31147 (6)	−0.0073 (2)	0.34153 (13)	0.0401 (5)	
H7	0.3369	−0.0687	0.3486	0.048*	
C8	0.30262 (6)	0.0842 (2)	0.26918 (12)	0.0354 (4)	
C9	0.26422 (6)	0.1752 (3)	0.25765 (13)	0.0419 (5)	
H9	0.2581	0.2361	0.2089	0.050*	
C10	0.23556 (7)	0.1743 (3)	0.31866 (13)	0.0431 (5)	
H10	0.2099	0.2342	0.3109	0.052*	
O6	0.45017 (16)	0.6539 (4)	0.3091 (3)	0.0555 (9)	0.719 (6)
O7	0.54917 (16)	1.1033 (5)	0.4276 (4)	0.0632 (11)	0.719 (6)
O8	0.59897 (13)	0.9699 (5)	0.5104 (3)	0.0758 (11)	0.719 (6)
H8A	0.6081	1.0636	0.5201	0.114*	0.719 (6)
C11	0.48910 (17)	0.6369 (5)	0.3610 (3)	0.0527 (11)	0.719 (6)
H11A	0.5100	0.5720	0.3332	0.063*	0.719 (6)
H11B	0.4836	0.5824	0.4130	0.063*	0.719 (6)
C12	0.50608 (12)	0.8096 (5)	0.3780 (2)	0.0552 (11)	0.719 (6)
H12A	0.5116	0.8619	0.3255	0.066*	0.719 (6)
H12B	0.4843	0.8742	0.4035	0.066*	0.719 (6)
C13	0.54702 (12)	0.8074 (4)	0.4348 (3)	0.0611 (12)	0.719 (6)
H13A	0.5415	0.7517	0.4864	0.073*	0.719 (6)
H13B	0.5688	0.7442	0.4084	0.073*	0.719 (6)
C14	0.5647 (2)	0.9760 (8)	0.4554 (5)	0.0500 (13)	0.719 (6)
O6B	0.4601 (3)	0.6099 (11)	0.3342 (8)	0.056 (3)	0.281 (6)
O7B	0.5371 (4)	1.1472 (15)	0.4022 (10)	0.079 (3)	0.281 (6)
O8B	0.5816 (5)	0.9780 (14)	0.4772 (8)	0.077 (5)	0.281 (6)
H8B	0.5954	1.0583	0.4958	0.115*	0.281 (6)
C11B	0.4986 (3)	0.5743 (13)	0.3876 (7)	0.052 (3)	0.281 (6)
H11C	0.5204	0.5281	0.3544	0.063*	0.281 (6)
H11D	0.4919	0.4925	0.4289	0.063*	0.281 (6)
C12B	0.5164 (3)	0.7285 (11)	0.4321 (6)	0.064 (3)	0.281 (6)
H12C	0.4938	0.7772	0.4627	0.076*	0.281 (6)
H12D	0.5400	0.6969	0.4725	0.076*	0.281 (6)
C13B	0.5326 (5)	0.8560 (13)	0.3745 (7)	0.086 (4)	0.281 (6)
H13C	0.5088	0.8878	0.3345	0.103*	0.281 (6)
H13D	0.5548	0.8061	0.3434	0.103*	0.281 (6)
C14B	0.5511 (5)	1.0101 (16)	0.4168 (9)	0.070 (5)	0.281 (6)
C15	0.42950 (8)	0.5072 (3)	0.28884 (15)	0.0543 (6)	
C16	0.39020 (8)	0.5418 (3)	0.24433 (16)	0.0583 (7)	
H16	0.3818	0.6508	0.2345	0.070*	
C17	0.36394 (7)	0.4158 (3)	0.21499 (14)	0.0481 (5)	
H17	0.3376	0.4389	0.1850	0.058*	
C18	0.37640 (6)	0.2527 (2)	0.22985 (12)	0.0373 (4)	
C19	0.41573 (7)	0.2172 (3)	0.27360 (13)	0.0452 (5)	
H19	0.4241	0.1080	0.2831	0.054*	
C20	0.44257 (7)	0.3452 (3)	0.30315 (14)	0.0530 (6)	
H20	0.4691	0.3225	0.3323	0.064*	

### Atomic displacement parameters (Å^2^)

**Table:**

	*U*^11^	*U*^22^	*U*^33^	*U*^12^	*U*^13^	*U*^23^
S1	0.0421 (3)	0.0388 (3)	0.0425 (3)	−0.0041 (2)	0.0004 (2)	−0.0036 (2)
O1	0.0396 (8)	0.0491 (9)	0.0560 (9)	0.0045 (7)	0.0085 (7)	0.0140 (7)
O2	0.0677 (11)	0.0382 (9)	0.0787 (12)	0.0006 (8)	−0.0076 (9)	0.0096 (8)
O3	0.0564 (10)	0.0474 (10)	0.0850 (12)	−0.0063 (8)	−0.0190 (9)	0.0033 (9)
O4	0.0603 (10)	0.0783 (12)	0.0377 (8)	−0.0082 (9)	−0.0060 (7)	0.0012 (8)
O5	0.0559 (10)	0.0374 (9)	0.0750 (11)	0.0022 (7)	0.0126 (8)	−0.0089 (8)
C1	0.0389 (11)	0.0539 (14)	0.0462 (12)	−0.0021 (10)	−0.0034 (9)	0.0087 (10)
C2	0.0507 (13)	0.0572 (14)	0.0459 (12)	−0.0099 (11)	0.0050 (10)	−0.0076 (11)
C3	0.0445 (12)	0.0394 (12)	0.0558 (13)	−0.0019 (9)	0.0106 (10)	−0.0012 (10)
C4	0.0415 (12)	0.0422 (12)	0.0444 (12)	−0.0025 (10)	0.0066 (9)	−0.0025 (10)
C5	0.0324 (10)	0.0319 (11)	0.0474 (11)	−0.0065 (8)	−0.0010 (9)	0.0031 (9)
C6	0.0397 (11)	0.0354 (11)	0.0447 (11)	−0.0013 (9)	−0.0058 (9)	0.0095 (9)
C7	0.0332 (10)	0.0347 (11)	0.0508 (12)	0.0021 (9)	−0.0057 (9)	0.0044 (9)
C8	0.0331 (10)	0.0315 (10)	0.0405 (10)	−0.0057 (8)	−0.0032 (8)	−0.0007 (8)
C9	0.0394 (11)	0.0386 (11)	0.0457 (12)	−0.0025 (9)	−0.0084 (9)	0.0092 (9)
C10	0.0334 (11)	0.0372 (11)	0.0571 (13)	0.0022 (9)	−0.0050 (9)	0.0101 (10)
O6	0.051 (2)	0.0397 (19)	0.073 (3)	−0.0119 (16)	−0.0064 (15)	0.0036 (16)
O7	0.056 (3)	0.048 (3)	0.081 (3)	−0.006 (2)	−0.0220 (18)	0.000 (2)
O8	0.082 (3)	0.0502 (15)	0.087 (3)	−0.0161 (15)	−0.0477 (19)	0.0065 (18)
C11	0.057 (3)	0.053 (3)	0.049 (3)	−0.012 (2)	0.008 (2)	−0.001 (2)
C12	0.052 (2)	0.053 (2)	0.059 (2)	−0.0150 (18)	−0.0059 (18)	0.0000 (19)
C13	0.061 (3)	0.044 (2)	0.075 (3)	−0.0112 (18)	−0.0126 (19)	0.0000 (19)
C14	0.058 (4)	0.048 (2)	0.042 (4)	−0.011 (3)	−0.009 (3)	−0.004 (3)
O6B	0.054 (6)	0.028 (4)	0.084 (8)	−0.009 (4)	−0.009 (5)	0.004 (4)
O7B	0.069 (8)	0.061 (7)	0.105 (10)	−0.006 (5)	−0.011 (6)	−0.003 (6)
O8B	0.107 (14)	0.047 (5)	0.069 (9)	−0.023 (8)	−0.042 (6)	−0.001 (5)
C11B	0.049 (6)	0.044 (6)	0.061 (7)	−0.022 (5)	−0.013 (5)	−0.001 (5)
C12B	0.048 (6)	0.073 (7)	0.070 (7)	−0.009 (5)	0.005 (5)	−0.007 (6)
C13B	0.100 (11)	0.084 (8)	0.074 (8)	−0.035 (8)	0.015 (8)	−0.011 (7)
C14B	0.079 (10)	0.092 (13)	0.036 (7)	−0.024 (10)	−0.005 (6)	0.002 (8)
C15	0.0582 (14)	0.0433 (14)	0.0644 (15)	−0.0223 (12)	0.0231 (12)	−0.0135 (11)
C16	0.0601 (16)	0.0348 (12)	0.0825 (18)	−0.0020 (11)	0.0216 (14)	0.0016 (12)
C17	0.0453 (12)	0.0410 (12)	0.0583 (14)	0.0002 (10)	0.0056 (11)	0.0098 (10)
C18	0.0347 (10)	0.0362 (11)	0.0413 (11)	−0.0035 (9)	0.0058 (8)	0.0018 (9)
C19	0.0405 (12)	0.0384 (12)	0.0557 (13)	−0.0010 (9)	−0.0008 (10)	0.0024 (10)
C20	0.0394 (12)	0.0610 (15)	0.0581 (14)	−0.0110 (11)	0.0003 (10)	−0.0038 (12)

### Geometric parameters (Å, °)

**Table:**

S1—O4	1.4383 (15)	C11—H11A	0.9700
S1—O5	1.4389 (17)	C11—H11B	0.9700
S1—C8	1.761 (2)	C12—C13	1.498 (4)
S1—C18	1.761 (2)	C12—H12A	0.9700
O1—C5	1.352 (2)	C12—H12B	0.9700
O1—C1	1.440 (2)	C13—C14	1.497 (6)
O2—C4	1.221 (3)	C13—H13A	0.9700
O3—C4	1.301 (3)	C13—H13B	0.9700
O3—H3	0.8200	C14—H8B	1.2897
C1—C2	1.505 (3)	O6B—C15	1.416 (7)
C1—H1A	0.9700	O6B—C11B	1.436 (8)
C1—H1B	0.9700	O7B—C14B	1.208 (12)
C2—C3	1.511 (3)	O8B—C14B	1.321 (11)
C2—H2A	0.9700	O8B—H8A	1.2384
C2—H2B	0.9700	O8B—H8B	0.8200
C3—C4	1.493 (3)	C11B—C12B	1.519 (7)
C3—H3A	0.9700	C11B—H11C	0.9700
C3—H3B	0.9700	C11B—H11D	0.9700
C5—C6	1.391 (3)	C12B—C13B	1.498 (7)
C5—C10	1.400 (3)	C12B—H12C	0.9700
C6—C7	1.390 (3)	C12B—H12D	0.9700
C6—H6	0.9300	C13B—C14B	1.510 (8)
C7—C8	1.384 (3)	C13B—H13C	0.9700
C7—H7	0.9300	C13B—H13D	0.9700
C8—C9	1.398 (3)	C15—C20	1.386 (4)
C9—C10	1.373 (3)	C15—C16	1.387 (4)
C9—H9	0.9300	C16—C17	1.364 (3)
C10—H10	0.9300	C16—H16	0.9300
O6—C15	1.375 (4)	C17—C18	1.390 (3)
O6—C11	1.413 (5)	C17—H17	0.9300
O7—C14	1.207 (8)	C18—C19	1.384 (3)
O8—C14	1.323 (7)	C19—C20	1.387 (3)
O8—H8A	0.8199	C19—H19	0.9300
O8—H8B	0.7586	C20—H20	0.9300
C11—C12	1.510 (5)		
			
O4—S1—O5	119.39 (10)	C13—C12—H12B	109.4
O4—S1—C8	107.79 (9)	C11—C12—H12B	109.4
O5—S1—C8	108.49 (10)	H12A—C12—H12B	108.0
O4—S1—C18	108.03 (10)	C14—C13—C12	113.5 (4)
O5—S1—C18	108.01 (10)	C14—C13—H13A	108.9
C8—S1—C18	104.08 (9)	C12—C13—H13A	108.9
C5—O1—C1	119.19 (15)	C14—C13—H13B	108.9
C4—O3—H3	109.5	C12—C13—H13B	108.9
O1—C1—C2	107.20 (18)	H13A—C13—H13B	107.7
O1—C1—H1A	110.3	O7—C14—O8	123.4 (5)
C2—C1—H1A	110.3	O7—C14—C13	124.7 (6)
O1—C1—H1B	110.3	O8—C14—C13	111.8 (6)
C2—C1—H1B	110.3	O7—C14—H8B	89.9
H1A—C1—H1B	108.5	C13—C14—H8B	145.3
C1—C2—C3	115.04 (18)	C15—O6B—C11B	132.5 (7)
C1—C2—H2A	108.5	C14B—O8B—H8A	134.2
C3—C2—H2A	108.5	C14B—O8B—H8B	115.4
C1—C2—H2B	108.5	O6B—C11B—C12B	111.5 (8)
C3—C2—H2B	108.5	O6B—C11B—H11C	109.3
H2A—C2—H2B	107.5	C12B—C11B—H11C	109.3
C4—C3—C2	115.44 (19)	O6B—C11B—H11D	109.3
C4—C3—H3A	108.4	C12B—C11B—H11D	109.3
C2—C3—H3A	108.4	H11C—C11B—H11D	108.0
C4—C3—H3B	108.4	C13B—C12B—C11B	113.9 (8)
C2—C3—H3B	108.4	C13B—C12B—H12C	108.8
H3A—C3—H3B	107.5	C11B—C12B—H12C	108.8
O2—C4—O3	123.0 (2)	C13B—C12B—H12D	108.8
O2—C4—C3	122.7 (2)	C11B—C12B—H12D	108.8
O3—C4—C3	114.26 (19)	H12C—C12B—H12D	107.7
O1—C5—C6	125.11 (18)	C12B—C13B—C14B	115.3 (10)
O1—C5—C10	114.95 (17)	C12B—C13B—H13C	108.5
C6—C5—C10	119.93 (19)	C14B—C13B—H13C	108.5
C7—C6—C5	119.33 (18)	C12B—C13B—H13D	108.5
C7—C6—H6	120.3	C14B—C13B—H13D	108.5
C5—C6—H6	120.3	H13C—C13B—H13D	107.5
C8—C7—C6	120.56 (18)	O7B—C14B—O8B	123.1 (12)
C8—C7—H7	119.7	O7B—C14B—C13B	123.7 (13)
C6—C7—H7	119.7	O8B—C14B—C13B	112.9 (13)
C7—C8—C9	120.04 (19)	O6—C15—C20	130.9 (3)
C7—C8—S1	119.87 (15)	O6—C15—C16	108.6 (3)
C9—C8—S1	120.02 (15)	C20—C15—C16	120.5 (2)
C10—C9—C8	119.64 (18)	C20—C15—O6B	107.2 (4)
C10—C9—H9	120.2	C16—C15—O6B	131.9 (5)
C8—C9—H9	120.2	C17—C16—C15	119.9 (2)
C9—C10—C5	120.49 (19)	C17—C16—H16	120.0
C9—C10—H10	119.8	C15—C16—H16	120.0
C5—C10—H10	119.8	C16—C17—C18	120.2 (2)
C15—O6—C11	114.4 (3)	C16—C17—H17	119.9
C14—O8—H8A	109.7	C18—C17—H17	119.9
C14—O8—H8B	70.7	C19—C18—C17	120.29 (19)
O6—C11—C12	106.4 (4)	C19—C18—S1	120.77 (16)
O6—C11—H11A	110.4	C17—C18—S1	118.93 (16)
C12—C11—H11A	110.4	C18—C19—C20	119.6 (2)
O6—C11—H11B	110.4	C18—C19—H19	120.2
C12—C11—H11B	110.4	C20—C19—H19	120.2
H11A—C11—H11B	108.6	C15—C20—C19	119.5 (2)
C13—C12—C11	111.3 (4)	C15—C20—H20	120.3
C13—C12—H12A	109.4	C19—C20—H20	120.3
C11—C12—H12A	109.4		
			
C5—O1—C1—C2	174.77 (17)	O6B—C11B—C12B—C13B	−65.6 (15)
O1—C1—C2—C3	53.5 (3)	C11B—C12B—C13B—C14B	−179.2 (11)
C1—C2—C3—C4	73.3 (3)	C12B—C13B—C14B—O7B	−120.5 (19)
C2—C3—C4—O2	12.6 (3)	C12B—C13B—C14B—O8B	53.2 (19)
C2—C3—C4—O3	−168.2 (2)	C11—O6—C15—C20	−7.8 (7)
C1—O1—C5—C6	−11.3 (3)	C11—O6—C15—C16	174.8 (4)
C1—O1—C5—C10	170.19 (18)	C11—O6—C15—O6B	13.3 (16)
O1—C5—C6—C7	−177.81 (18)	C11B—O6B—C15—O6	−164 (3)
C10—C5—C6—C7	0.6 (3)	C11B—O6B—C15—C20	−0.3 (17)
C5—C6—C7—C8	0.2 (3)	C11B—O6B—C15—C16	172.4 (11)
C6—C7—C8—C9	−0.8 (3)	O6—C15—C16—C17	178.3 (3)
C6—C7—C8—S1	176.31 (15)	C20—C15—C16—C17	0.6 (4)
O4—S1—C8—C7	160.76 (16)	O6B—C15—C16—C17	−171.4 (8)
O5—S1—C8—C7	30.16 (18)	C15—C16—C17—C18	0.2 (4)
C18—S1—C8—C7	−84.68 (17)	C16—C17—C18—C19	−0.8 (3)
O4—S1—C8—C9	−22.18 (19)	C16—C17—C18—S1	177.98 (17)
O5—S1—C8—C9	−152.77 (16)	O4—S1—C18—C19	−146.43 (17)
C18—S1—C8—C9	92.39 (17)	O5—S1—C18—C19	−16.0 (2)
C7—C8—C9—C10	0.4 (3)	C8—S1—C18—C19	99.18 (18)
S1—C8—C9—C10	−176.62 (15)	O4—S1—C18—C17	34.8 (2)
C8—C9—C10—C5	0.4 (3)	O5—S1—C18—C17	165.25 (17)
O1—C5—C10—C9	177.64 (18)	C8—S1—C18—C17	−79.57 (19)
C6—C5—C10—C9	−1.0 (3)	C17—C18—C19—C20	0.5 (3)
C15—O6—C11—C12	−178.5 (4)	S1—C18—C19—C20	−178.27 (16)
O6—C11—C12—C13	178.5 (4)	O6—C15—C20—C19	−178.0 (4)
C11—C12—C13—C14	−178.6 (4)	C16—C15—C20—C19	−0.9 (4)
C12—C13—C14—O7	−2.6 (9)	O6B—C15—C20—C19	172.8 (6)
C12—C13—C14—O8	175.4 (5)	C18—C19—C20—C15	0.4 (3)
C15—O6B—C11B—C12B	−171.3 (12)		

### Hydrogen-bond geometry (Å, °)

**Table:**

*D*—H···*A*	*D*—H	H···*A*	*D*···*A*	*D*—H···*A*
O8B—H8B···O2^i^	0.82	2.07	2.890 (11)	180
O8—H8A···O2^i^	0.82	1.77	2.587 (4)	178
O3—H3···O7B^ii^	0.82	1.84	2.623 (13)	159
O3—H3···O7^ii^	0.82	1.85	2.668 (5)	177

Symmetry codes: (i) *x*+1/2, *y*+3/2, *z*; (ii) *x*−1/2, *y*−3/2, *z*.
